# Our Summer at Maplewood, Chap. IV., Food and Fancy

**Published:** 1875-05

**Authors:** 


### CHAPTER IV.

### FOOD AND FANCY.

EMELINE passed her plate for a second piece of chicken, remarking, as she did so, "These chickens that you and Alice fry must be a new kind, for so far as I can discover, they have neither backs, necks, legs, nor wings. Truly a valuable breed this must be, which runs entirely to breast and second joints; the frying kind, I suppose. But seriously, Kate, what do you do with the remainder of these fowls, and how do you cut such plump, compact little pieces, each one of which is a delicious surprise on a near acquaintance?" I was thinking about another matter, and replied, "Cousin Emeline, in this cook-book of mine I mean to devote one whole chapter to the consideration of suitability. I have a friend, the charming Mrs. Rose; you used to know her; a woman of fine native talent, rare culture, easy, affable manner, and great personal beauty. But her one charm, which with me outshines all others, is tact. She has tact more perfectly developed than any one I ever knew. Prince or beggar feels alike at ease in her presence; but she would never think of inviting them to her house on the same occasion. If she is going to have a little supper party of ten or a dozen, and her guests are invited to meet a friend who may be staying with her, she says to herself, 'Now here is Fanny with such and such tastes. She is this order of person. I will invite those people to meet her who are harmonious; such as will call out her peculiar powers; some sharp contrast. She goes to work in an artistic manner and selects her guests, having reference mainly to suitability. This same tact is called into exercise in deciding what her dinner or supper shall be. The principal dish determines what shall be its companions. But many women seem to lose sight entirely of this suitability of things, especially in eating. Such are just as likely to have griddle-cakes and fish for breakfast, as griddle-cakes and stewed chicken, or griddle-cakes and beefsteak."

"But to return to this chicken," said Emeline. "In all my travels, at home and abroad, I never tasted its equal. How is it prepared?"

"Why, you see, mamma," said Alice, "having this fried chicken for dinner necessitates having stewed chicken for breakfast, either before or after, in order to use the other parts. But this is true economy, Cousin Kate says, for while the breast and second joints are best fried, the other parts are much better stewed. From the breast we got three nice frying pieces, so nearly alike that you can scarcely tell them apart. Having decided where to cut it, instead of sawing and haggling with a dull knife, and in the struggle mutilating the pieces so that they remind you of 'the ragged edge of despair,' Cousin Kate puts the breast of the chicken on the meat-board, and placing the knife just where she wishes to cut, strikes a blow that sends the blade through the chicken at one clean stroke. Having washed the pieces and dried them on a soft towel, while the lard, of which there should be a liberal supply, is heating, rub each piece of chicken lightly, with salt, cayenne and black pepper, which has been mixed together in suitable proportions, and just before dropping into the boiling lard, dust it with flour. To ascertain if the lard is hot enough, slip into it a slice of raw potato, and when the potato is brown, the lard is sufficiently heated. Drop into the boiling lard one piece of chicken at a time, and let the bubbling and tumult, incident to its reception, somewhat subside before adding another. When the pieces are of a rich brown color, they will be found well cooked, and may be placed on a tin or wire strainer in a warm oven, until ready to serve. Chicken fried in this manner is much less greasy than when cooked in a small quantity of lard. Nearly all kinds of fresh fish are best fried in the same way. To parboil a chicken before frying, Cousin Kate says, is barbarous treatment, not of the chicken but of the people who eat it after it has been robbed of its best juices."

"Now, Alice, tell me how you make these Graham 'Gems,' for I really think they deserve the name."

"To a pint each of cold, sweet milk and pure water, add a teaspoonful of sugar, a saltspoonful of salt, and three pints of Graham flour. Sprinkle in the flour slowly, stirring the mixture rapidly, and when all is added beat the dough briskly for a minute or two before an open window or door; for Cousin Kate says, pure air is very essential to the life of bread, especially bread which is made light by beating. Bake the gems in iron gem-pans or puff-pans, half filling the cups which must be well heated and greased. Bake in a quick oven, the greatest heat being applied to the bottom when first put into the oven.

"Fine flour gems, or English muffins are made in the same manner as Graham, omitting the sugar, and adding a half pint of flour. Gems are good made with water instead of milk and water.

"A very excellent Graham bread is made by putting a teaspoonful of sugar and a saltspoonful of salt into a quart of Graham flour and pouring upon it boiling water, stirring it meanwhile until all is wet and soft enough to be worked into a mass resembling a large roll, or small loaf of bread. Work it as little as possible, and when brought into this shape, cut it with a sharp knife, dipped in flour, into slices half an inch thick. Place these in a bread-pan and bake in a hot oven for half an hour, or until brown. There, Cousin Kate, haven't I said my lesson well?"

"Perfectly. And having induced you, Alice, to make and taste these kinds of Graham bread, I am content, well knowing that you will push your investigations, and find by experiments that the varieties of delicious bread which can be made of Graham flour and water are almost numberless."

At breakfast next morning the stewed chicken was a failure, at which Alice was much mortified. She said, deprecatingly:

"Cousin Kate, it is not at all like yours, yet I was very careful to read up my notes, which said 'Two chickens, minus the frying pieces, are equal to one whole chicken. To stew them, add one half pint water, cover closely and boil gently or simmer smartly, until very tender. Then drain from them the water, add a piece of butter, or the oil from the water you have drained out, let them brown nicely, turning the piece when necessary.' That is what is the matter, Cousin Kate, I didn't brown the chicken. Who would suppose so small a thing would make so vast a difference?"

"This browning, Alice, changes the whole character of the dish, and makes rich and appetizing what is otherwise weak and vapid."

"Go on Alice," said her mother, "give me the rest of the recipe."

"When browned, lift the chicken, and pour into the stew-pan the water previously drained out, having removed any surplus oil. When it boils, thicken with flour, stirred to a smooth paste in a little cold milk, or sweet cream.

Make it thick enough to deserve the name of gravy, season with salt and pepper, and return the chicken to it, letting it remain in the stew-pan long enough to become thoroughly saturated. Dish altogether."

"Kate," remarked Emeline, "I have been prejudiced all my life against stewed chicken, because the gravy was so inefficient, such a wretchedly demoralized mixture, tasting a little of chicken and very much of water and raw flour. But stewed chicken *a la* Cousin Kate, has my hearty approval."

"Beef, veal, and lamb, are all delicious when stewed in the same manner," I said to Alice. Then Cousin Emeline continued :

"Kate, I wonder why it is that women abhor the kitchen, and so detest pottering among the pots and pans ? You open this subject to me in a new light, and I lose sight of the drudgery and unpleasant work in my interest about results, and my enquiries after the cause. When we consider, there is nothing of greater importance than this question, What shall we eat, and how shall it be cooked ? For what order of men and women we are, depends very much, Scientists say, upon the food we eat. The world seems divided into two classes, mainly ; those who live to eat, and those who regard eating a troublesome and expensive necessity, but a nuisance. There are very few who eat to live in the truest sense."

"The trouble, Cousin Emeline, is that people don't think ; they go stumbling and groping through life because they haven't found out that their brains are for use. They never consider or think about these things. If mothers realized that the food their children eat has a positive quality, that it actually contains the principle of life or death, health or disease, they could not be so indifferent. Did women realize the vital importance of this matter, they would no sooner trust to a slovenly, unskilled cook the preparation of their children's food, than they would the washing of their expensive laces. Do they think more of their honitons and valenciennes than of their Toms and Marys ? I think not. But one washing of a fine lace by a careless Bridget, reduces it to useless rags, whereas, these living and breathing jewels are destroyed more slowly, and the mother dees not see the wear and tear of bad food so palpably."

---

One evening Emeline and I were sitting on the western veranda watching the sunset, when Alice ran lightly up the steps. Her hat was in her hand, and her beautiful hair, unconfined by comb or plait, floated at will in wavy splendor. Her cheeks were glowing and her eyes sparkling as she spoke excitedly :

"Oh, mamma, I've had an adventure this evening. The air was exhilarating, the birds sang so gaily, and all nature seemed so blithe and happy, that I, too, took wing, and flew, rather than walked, this way and that, unheeding, in truth not caring, where the lovely crooked ways led me ; until suddenly I came upon a small cottage by the roadside. It was nestled just under the hill, and was a very bird's-nest for coziness. I came upon it from the rear, through a garden all aglow with red roses and carnation pinks, and had almost walked into the house before I realized what I was doing. The door stood open, and looking out at me amazed, as well they might be, was a pair of large brown eyes. So earnest and beseeching was their gaze that I stood spell-bound, like one dazed or in a dream. The owner of these marvelous eyes was a young girl who seemed an invalid, as she reclined on a lounge beside which the small supper table was placed. Another lady sat at the table with her back toward me. A look or low spoken word from the sick girl directed this lady's attention to me, and she came out and invited me in to rest. I didn't care to rest, but I did care to learn something of this brown-eyed beauty who seemed sick and sad. But no, I think I am wrong there ; her eyes only had a wistful look, as if asking, pleading for something they dared not hope to gain ; but her voice was cheerful and her laugh free and joyous. Yet she suffers a great deal and has been for years confined to her lounge by some affection of the spine. Mamma, I think you made a mistake in writing your novel before I discovered this lovely heroine."

Emeline responded—

"By the way, Kate, did I tell you of 'My Lord' and his mother whom we met last summer in Switzerland ? We talked with them upon several occasions, yet did not learn their name or the land of their nativity. I thought them Americans from their manners and speech, but Alice insisted that they were English. She imagined him to be an English lord, 'the last of his race,' and that, she thought, accounted for their deep mourning and great dejection."

"No," I answered, while a vague suspicion crossed my mind, "you have never spoken of them to me, but I am interested, Describe them more fully. What were they like in personal appearance ?"

"Very much like each other, both having the same dark, deep eyes—eyes that remind you of bottomless wells of pure water, with shadows lingering about them hinting of sunshine. He was 25 or 30 years of age, and she old enough to be his mother. His hair was dark, and rippled or waved back from his forehead, while hers was so white that it reminded one of a fleecy cloud floating about her face."

"Was she slightly lame ?" I asked.

"Yes," answered Emeline, "how came you to guess *that* grace belonged to her ?"

"Perhaps my question was the offspring of your remembrance," I answered evasively, continuing. "And Alice thought him a hero, did she ? And in her visions of romance she has no doubt visited his ancestral home, his lordly domain ? How is it, my dear, do you dream of him still ?"

A rosy flush swept over her fair face as she answered :

"Indeed, Cousin Kate, I never dreamed of him but once in my life, and that was the first night I slept in this house. You remember it was after dark when we arrived, and to me it did seem a little wierd and dismal for three lone women to take possession of a roomy old mansion like this, and although I wouldn't own it then, I was nervous and fidgety, starting at every sound all the evening. That I should dream strange dreams that night was a matter of course."

"But I never heard you mention this fact before Alice," said her mother. "Pray tell us this strange dream "

"I would rather not," said Alice, blushing more deeply, "lest you think me a silly girl, and vain as foolish."

By this time Alice had really aroused our curiosity, and Cousin Emeline and I both protested that dreams were only dreams, and that we should not think of attaching any importance whatever to hers. Finally we induced her to tell us her dream, in which she said she rehearsed the scene of our arrival at Maplewood with this variation : she had lost sight of her mother and me, and entered the wide gloomy hall alone.

As she stepped timidly forward, she heard the great door close with a clang behind her, shutting her, like a prisoner, within. A wild terror seized her, and she was about to cry out for help, when a voice of exceeding sweetness said gently, "Welcome home, my darling." "I knew the voice," said Alice, "and looking up beheld 'My Lord' descending the hall stair. But the curious part of the dream was that he bore in his arms a beautiful child, laughing and crowing with delight."

"Could that have been the boy Cupid, I wonder?" said I, teasingly, while Emeline seemed interested, and asked:

"What happened next, Alice? What did you say or do?"

"I guess I ran away, for I remember no more."

A few days after this conversation, I sat at a table in the large old kitchen beside Alice, who was earnestly engaged writing out the recipe for buns. The summer breeze played idly with her brown curls, while the delicious odor of the freshly-baked buns mingled with that of the wild rose and honeysuckle, making a confusion of sweet smells. At length, laying down the pen, Alice said:

"Cousin Kate, I want to take some of these airy things (the buns) to Jessie James this evening, with a basket of the brightest, sweetest strawberries Mike can find in all the garden. May I?"

I bowed assent, and she went on:

"Jessie knows all about your friends, the Douglasses, and has promised to tell me about his beautiful young wife, whose sudden death almost broke the heart of her husband."

Looking up from my work, I said:

"*Whose* beautiful young wife? What *are* you talking about, Alice?"

"About Gerald Douglass, whose wife died so suddenly just before they went abroad. Jessie says that was the reason of their going, and that his mother is devoted to him, and would go with him to the ends of the earth if he desired."

"Very likely," I replied, "most mothers would do the same."

"But why, Cousin Kate, did you never tell me of this only son of your old friend?"

"Why, bless me, child, when and why, pray, should I have told you the history of this particular young man? Have you not been for years abroad, and before that, close shut within the walls of the 'Sacred Heart,' conning your lessons ever since you were a child? Never until now, Alice, did I realize what a fearful responsibility rests upon a maiden lady whose old friend and schoolmate happens to have a terrible only son. Ah, woe is me!"

"Now, Cousin Kate, please don't laugh at me, but shut up here with only your cook-book and mamma's novel to think about, is it strange that I like to hear Jessie tell of these people? Indeed, I think it would be very ungrateful not to be interested in them, since we've taken possession of their beautiful home and all their lovely things, just as if they belonged to us. Jessie says that the family portraits are in the drawing-room, and that the suite of rooms in the north wing belonged to his wife, and were fitted up according to her taste when she came here, a bride; and that they are kept just as she left them. Would you mind asking Susanah to show them to us sometime, when she opens to air and dust them; or do you think she would object?"

"Oh, no! Susanah will be only too glad to show all her treasures, and I will ask her some day. But we must give some attention to the realities of the culinary department before we wander into the fields of romance."

